# Modeling the impact of genetically modified male mosquitoes in the spatial population dynamics of *Aedes aegypti*

**DOI:** 10.1038/s41598-022-12764-7

**Published:** 2022-06-01

**Authors:** Monalisa R. da Silva, Pedro H. G. Lugão, Fábio Prezoto, Grigori Chapiro

**Affiliations:** 1 Laboratory of Applied Mathematics (LAMAP), Federal University of Juiz de Fora, Juiz de Fora, MG Brazil; 2 Computational Modeling Graduate Program, Federal University of Juiz de Fora, Juiz de Fora, MG Brazil; 3 Federal Institute of the Southeast of Minas Gerais, Santos Dumont, MG Brazil; 4 Computational Modeling Graduate Program - National Laboratory for Scientific Computing (LNCC), Petrópolis, RJ Brazil; 5 Department of Zoology, Institute of Biological Sciences, Federal University of Juiz de Fora, Juiz de Fora, MG Brazil

**Keywords:** Ecological modelling, Population dynamics, Applied mathematics

## Abstract

The mosquito *Aedes aegypti* is the primary vector of diseases such as dengue, Zika, chikungunya, and yellow fever. Improving control techniques requires a better understanding of the mosquito’s life cycle, including spatial population dynamics in endemic regions. One of the most promising techniques consists of introducing genetically modified male mosquitoes. Several models proposed to describe this technique present mathematical issues or rely on numerous parameters, making their application challenging to real-world situations. We propose a model describing the spatial population dynamics of the *Aedes aegypti* in the presence of genetically modified males. This model presents some mathematical improvements compared to the literature allowing deeper mathematical analysis. Moreover, this model relies on few parameters, which we show how to obtain or estimate from the literature. Through numerical simulations, we investigate the impacts of environmental heterogeneity, the periodicity of genetically modified male releases, and released genetically modified males quantity on the population dynamics of *Aedes aegypti*. The main results point to that the successful application of this vector control technique relies on releasing more than a critical amount of modified males with a frequency exceeding a specific critical value.

## Introduction

Dengue is considered one of the main sources of public health problems in the world by the World Health Organization (WHO)^1^. Among vector-borne diseases, dengue is the fastest-spreading worldwide^2^. In the last century, especially during the 1950s and 1960s, vector control programs in many countries with relative success used chemical strategies without restriction on the use of insecticides. In the last 50 years, this endemic disease has grown 30 times, expanding geographically to new countries and, in the present decade, has been concentrated in urban environments^3^.

Campaigns against dengue at the end of the last century already prioritized the educational process, sanitation, and the use of chemical control restricted to moments of epidemics. However, the control measures of the dengue-transmitting mosquito *Aedes aegypti* (Linnaeus, 1762) did not present a significant effect mainly due to its high reproductive capacity and genomic flexibility that can be seen in two aspects. First, the rapid selection of populations resistant to chemical and biological insecticides. Second, due to the existence of a variety of closely related species, forming complexes of cryptic species (species that are so similar that they are thought to be representatives of one^4^), some of which appear to be undergoing speciation in the process of adapting to the environment modified by man^5^. The mosquito *Ae. aegypti* is a synanthropic and anthropophilic species. In recent decades it has proliferated with great success in urban centers, primarily due to human behavior itself, which ends up providing and neglecting possible breeding sites for their reproduction^6^. It can be said that *Ae. aegypti* is mainly distributed in urban and suburban areas, where anthropic changes favor its proliferation^7^.

In search of more viable strategies, genetic manipulation has been introduced in an attempt to reduce the population of *Ae. aegypti*. This method uses the release of males carrying a lethal gene, producing the tTAV protein, causing death, before the adult stage of the descendants of male genetically modified (GM) mosquitoes and wild females^8^. OX513A is a RIDL (Release of Insects carrying a Dominant Lethal) strain that carries a repressible dominant lethal transgene insertion causing lethality at a late larval, or early pupal stage^9^. The mating progeny between transgenic males and wild females inherits a copy of the OX513A insert and, consequently, dies before adult metamorphosis^9^.

British company Oxford Insect Technology (Oxitec) develops genetically modified individuals for the *Ae. aegypti* mosquito. Tests were carried out using the OX513A strain in some places that have high dengue rates, such as Malaysia and the Cayman Islands in 2010, with a significant reduction of the wild *Ae. aegypti* mosquito^8,10,11^. The use of the OX513A strain was also applied successfully in Brazil^8,12^.

Despite the growth in the use of genetically modified individuals to combat dengue, population modeling strategies are necessary to understand the dynamics involved and thereby estimate the most appropriate release moment for the effectiveness of the mosquito control strategy. For example, the employment of RIDL mosquitoes is reported to be ecologically friendly and effective^13^. However, it presents elevated operation costs, deal with significant amount of mosquitoes among other application limitations^13^, which can be attenuated by applying only the necessary quantities of mosquitoes motivating the present research.

There are several strategies for modeling the mosquito population dynamics in the literature. Among them, mathematical models based on ordinary differential equations (ODE) are used^14,15^ to study the importance of temperature and precipitation in mosquito population patterns; also in the context of genetically modified mosquitoes release^16^. This approach possesses natural limitations, as it can not account for environmental heterogeneity, e.g., the difference between house blocks and streets. As another example, GM mosquito application is made in some localized releases. Other studies^17^ use graph theory to extend ODE modeling to two dimensions. This approach presents difficulties due to the excessive number of parameters, which have to be determined.

The hypothesis that mosquito’s erratic movement is similar to the diffusion process leads to models based on partial differential equations (PDEs)^18–22^. Yamashita et al.^21^ studied the population dynamics of *Ae. aegypti* using a mathematical model based on a diffusion-convection-reaction system. Only two subpopulations were considered, a winged phase (mature female) and an immobile phase (eggs, larvae, and pupae). Other similar models were studied in the literature^18,21,23^.

The present study intends to extend this population dynamics model in order to make it more realistic and to include the impact of the GM male *Ae. aegypti* mosquitoes as a control strategy of dengue. We investigate the impacts of heterogeneity, GM males release periodicity and released GM males quantity on the population dynamics of *Ae. aegypti*.

## Modeling

In the present work, we extend the base model for the spatial mosquito population dynamics^24^ to include wild male mosquitoes and genetically modified male mosquitoes. Thus, five populations will be considered: the aquatic mosquito population, including larvae and pupae, the egg mosquito population, the reproductive female mosquito population, the wild male mosquito population, and the genetically modified male population. Similar approaches can be found in the literature^25,26^.

In the following system, we represent mosquito population densities (mosquitoes per m$^2$) by: *E* - in the egg phase, *A* - in the aquatic phase, *F* - female in the reproductive phase, *M* - wild males, and *G* - genetically modified male mosquitoes. Due to the very high resistance of the egg phase (up to 450 days^27^) and as we are interested in an urban spatial macro-scale modeling, we do not consider the mortality in the egg phase. The model is described by the following system of partial differential equations:

1 $$\begin{aligned} {\left\{ \begin{array}{ll} \partial _t E &{} = \alpha \beta F M -e E, \\ \partial _t A &{} = e \left( 1 - \dfrac{A}{k} \right) E -(\eta _a+{\mu _a})A, \\ \partial _t F &{} = \nabla \cdot (D_m \nabla F) -\mu _f F + r\eta _{a} A, \\ \partial _t M &{} = \nabla \cdot (D_m \nabla M) -\mu _m M + (1-r)\eta _{a} A, \\ \partial _t G &{} = \nabla \cdot (D_g \nabla G) -\mu _{g}G + l, \end{array}\right. } \end{aligned}$$

where $\alpha$ represents the proportion of wild male mosquitoes to the total number of male mosquitoes (wild males + genetically modified males); $\beta$ represents the expected quantity of eggs from the successful encounter between wild females and males; *e* is the egg hatching rate; *k* is the carrying capacity of the aquatic phase; $\eta _a$ is the emergence rate for mosquitoes from the aquatic phase to the female or male phases; $\mu _a$, $\mu _f$, $\mu _m$, and $\mu _{g}$ are the mortality rates of mosquitoes in the aquatic phase, females, males, and genetically modified males, respectively; *r* is the proportion of females to males (typically $r=0.5$); $l=l(x,y,t)$ is the function representing the number of genetically modified mosquitoes released in a unit of time at any point of the domain; $D_m$ is the diffusion coefficient of wild mobiles females and males; $D_g$ is the diffusion coefficient of genetically modified males. The proposed model (1) can naturally deal with heterogeneous parameters, such as mortality, diffusion, and carrying capacity coefficients. Thus it is possible to model the influence of rain, wind, and human action. In the context of this work, we are considering that the city neighborhood is divided into two environments: houses and streets. Due to lack of data, we restrict the investigated heterogeneity only to the carrying capacity coefficient.

The proposed model can be regarded as an extension of other “economic” models^20,24^ in the effort to qualitatively reproduce the complex phenomena by using as few parameters as possible. Following this idea, the carrying capacity was neglected in the egg phase because of the skip oviposition phenomenon^28^ i.e., the female lays the number of eggs that the place holds, without more space, she migrates to other environments to finish laying the eggs. We also do not consider this coefficient in the winged phase as limitations in the winged phase were not reported in any study. On the other hand, we consider it in the aquatic phases (larvae and pupae), where it is effective^29^.

The term $\alpha$, which multiplies the probability of encounters between male and female, represents the impact of the insertion of genetically modified males in the mosquito population to the immobile phase and is defined as

2 $$\begin{aligned} \alpha = \left\{ \begin{array}{cc} 1, &{} \text{ if } M=G= 0, \\ \dfrac{M}{M + G}, &{} \text{ otherwise }. \end{array} \right. \end{aligned}$$

Similar modeling approach can be found in the literature^16^. As the release rate of genetically modified males increases, the alpha value decreases, and, consequently, the probability of encounter between females and wild males also decreases. Thus, there is a greater probability of encounter between genetically modified males and females. This approach presents an advantage, when compared to the models found in the literature^25^, as System  (1) does not present singularities at the equilibrium states, allowing mathematical analysis and numerical simulations. From the biological point of view, the increment of male wild mosquitoes over some critical value does not affect the egg deposition. At first glance, the term *FM* can lead to a misunderstanding that such property is not satisfied in the presented model. However, in Section “Equilibrium points considering the application of genetically modified male mosquitoes,” we argue that both male and female populations possess mathematical attractor equilibria, blocking the wild male population from growing beyond this value.

Finally, any acceptable population model should be invariant in the definition domain, meaning its solution does not present senseless values. Setting the variable domain as

3 $$\begin{aligned} 0 \le E(x,y,t)< \infty ,\;\; 0 \le A(x,y,t) \le k, \;\; 0 \le F(x,y,t)< \infty ,\;\; 0 \le M(x,y,t)< \infty ,\;\; 0 \le G(x,y,t) < \infty , \end{aligned}$$

we can verify that it is invariant under the time evolution by the System (1). To prove this statement, it is sufficient to verify that the vector field defined by the right side of (1) points into the domain when (*E*, *A*, *F*, *M*, *G*) approaches the domain boundary.

- When *E* approaches zero, the right side of the first equation in (1) is not negative.
- When *A* approaches zero, the right side of the second equation in (1) is not negative. When *A* approaches *k* (bottom), the first term on the right side of the second equation in (1) tends to zero, while the second term remains negative.
- Since the term $\nabla \cdot (D_m \nabla F)$ cannot change the *F* sign, when *F* approaches zero, the right side of the third equation in (1) is not negative
- Since the term $\nabla \cdot (D_m \nabla M)$ cannot change the *M* sign, when *M* approaches zero, the right side of the fourth equation in (1) is not negative.
- Since the term $\nabla \cdot (D_g \nabla G)$ cannot change the *G* sign, when *G* approaches zero, the right side of the fifth equation in (1) is not negative.

In the rest of this section, let us explain how to estimate one-by-one all the parameters used in this model from experimental data available in the literature. It is a challenging task as, typically, the development of the *Ae. aegypti* mosquito depends on food variation^30^, temperature variations^14,15^ and rainfall^31^. This data is not available in the literature in the organized and systematic form. Because of that, we assume the environment is under optimal conditions of temperature, availability of food, and humidity.

### How to estimate emergence rate $(\eta _a)$

The emergence rate describes the rate at which the aquatic phase of the mosquito emerges into the adult phases. In the present model, for simplicity, it was considered that no mosquito from the crossing between genetically modified males and females reaches adulthood. Thus, the emergence rate is calculated on the crossing between females and wild males. Under optimal conditions and feeding distribution, based on the literature^30^, the emergence rate is 0.5596 $\text{ day}^{-1}$.

### How to estimate diffusion coefficients $(D_m,D_g)$

The diffusion coefficient is one of the most important parameters describing the mosquitoes’ movement. We use the methodology proposed in the previous work^24^ to obtain the diffusion coefficient of adult mosquitoes (females and males) and genetically modified males.

The estimate is done by assuming that all mosquitoes are released at (0, 0), and their movement is described by the corresponding equation in (1) neglecting other terms than diffusion. The population starts spreading in all directions. We define the spreading distance *R*(*t*) as the radius of the region centered in (0, 0) where $90\%$ of the initial mosquitoes population density is present. In Silva et al.^24^ it is shown that

4 $$\begin{aligned} R(t) = \sqrt{4Dt} \;\text {erf}^{-1}(0.9). \end{aligned}$$

Now corresponding diffusion coefficient is estimated by using the average flight distance of the mosquitoes and the characteristic time related to their life expectancy. Under favorable weather conditions, the average lifetime flight distance of females and males is approximately^32,33^ 65 m, while the same for GM males is^34^ 67.3 m. Based on the literature, we consider that the characteristic time for wild females and males^32^ is 7 days, and the same for genetically modified males is^34^ 2.17 days. Using (4) we estimate the values for $D_m$ and $D_g$ summarized in Table 1. It would be natural to consider that the mosquitoes’ movement changes in different environments. Unfortunately, we were unable to find the corresponding experimental data, and because of that, we considered that $D_m$ and $D_g$ are the same in streets and house blocks.

### How to estimate mortality rates ($\mu _a$, $\mu _f$, $\mu _m$, $\mu _{g}$)

The mortality coefficient represents an average quantity of mosquitoes in the corresponding phase dying each day. As mentioned before, we disregard the mortality rate in the egg phase, as it is negligible due to its great durability^27^, it does not affect the numerical results, and it complicates analytical estimates. Thus, the aquatic phase mortality rate coefficient is equal to the same for larvae’s coefficient, which is approximately^29^ $\mu _a = 0.025$ (1/day).

There is no solid agreement on the mortality rate of male and female wild mosquitoes in the literature. Although some results^29,30^ suggest they are similar, we follow these authors and consider them equal. Considering both natural death and accidental ones, approximately $10\%$ of females and male mosquitoes in the adult phase die at each day^35^. Under optimal conditions, the mortality coefficient can be estimated from this data by using the proposed model (1) by neglecting diffusion and emergence terms in the corresponding equation; details can be found in the previous work^24^. The resulting parameter values are summarized in Table 1.

It would be natural to consider that the mosquitoes mortality rate depends on the environment. Unfortunately, we were unable to find the corresponding experimental data, and because of that, we considered that $\mu _a$, $\mu _f$, $\mu _m$, and $\mu _{g}$ are the same in streets and house blocks.

### How to estimate the expected egg number ($\beta$)

This coefficient represents the average quantity of eggs a wild female lays per day, assuming a successful meeting with a wild male. Considering the number of times a female lays eggs in its lifetime^36^, the average quantity of eggs per lay and the mosquito’s life expectancy, under favorable conditions, this coefficient is estimated as $\beta = 34$.

### How to estimate the hatching rate (*e*)

This coefficient determines the average number of eggs hatching in one day. Experimental data^37^ suggest that, under optimal humidity conditions, the mean value of the hatch rate coefficient is 0.24 given a temperature of 28 ($^{\circ }$C), which is considered ideal for mosquito development. This is the value used in the present work.

### How to estimate carrying capacity coefficient (*k*)

The carrying capacity *k* represents the space limitation of one phase due to situations present in the environment^37,38^, such as competition for food among the larvae^39^. In general, it depends on external factors such as food availability, climate, terrain properties, making direct estimation almost impossible. In the Analytical results section, we show how to estimate this coefficient for each grid block. When considering spatial population dynamics in a heterogeneous environment, carrying capacity is one of the most influential parameters as it varies significantly. For example, house block offer more food and a shelter against natural predators resulting to a larger carrying capacity when compared with street environment. Following the literature^32^ we assume that the 80% of the mosquito’s breeding places are in houses resulting in the relation $k_h=5k_s$, where $k_h$ and $k_s$ are the carrying capacities of the house blocks and in the streets.

### Genetically modified mosquitoes release rate (*l*)

Function *l*(*x*, *y*, *t*) determines how many genetically modified mosquitoes are released in the location (*x*, *y*) at time *t*.

In a normal situation, the sex ratio between males and females is 1 : 1. The increment of this proportion favoring GM males increases the probability of females to mate with these mosquitoes. As reported in the literature^12,30^ the initial launch size is 11 times larger than the adult female population, and it is done in some spots in the city. In this work, we analyze different release strategies maintaining the $11\times 1$ proportion in some scenarios.

**Table 1:** All parameter values are directly taken or estimated from the literature as explained in section Modeling.

Parameter	Description	Value	Sources
$D_m$	Female and Male diffusion coefficient	111 (m$^2$/day)	Fitted^32^
$D_g$	Male GM diffusion coefficient	331.4062 (m$^2$/day)	Fitted^34^
$\mu _f$	Female phase mortality rate	0.1177 (1/day)	^24^
$\mu _m$	Male phase mortality rate	0.1177 (1/day)	^24^
$\mu _{mg}$	Male GM phase mortality rate	0.6200 (1/day)	^30^
$\mu _a$	Aquatic phase mortality rate	0.0250 (1/day)	^29^
$\eta _a$	Emergence rate	0.5596 (1/day)	^30^
*e*	Hatching rate	0.2400 (1/day)	^37^
$\beta$	The expected egg number	34 (m$^2$/day)	^36^
$k_s$	Carrying capacity (streets)	0.1402 (1/m$^2$)	Fitted
$k_h$	Carrying capacity (house blocks)	0.0280 (1/m$^2$)	Fitted

## Analytical results and discussion

### Simplifying the model to total population dynamics

In order to estimate the carrying capacity coefficient *k* we follow previous work^24^ and transform the spatial population dynamic model written in terms of PDEs into a total population dynamic model written in terms of ODEs.

Let $\chi \in \mathbb {R}^2$ be a small part of the domain, where the variables *E*, *A*, *F*, *M* and *G* can be considered homogeneous. In our case, we considered one cell in a discretized domain. Notice that with limitations in the experimental data, where there are always a limited number of pitfalls, the assumption on local homogeneity is correct. Considering $\chi$ to be a compact set with smooth boundary $\Gamma$, and that functions *F*, *M*, *G* are sufficiently smooth, Gauss’s Theorem results in:

5 $$\begin{aligned} \displaystyle \iint _{\chi } \nabla \cdot (D_m \nabla F) dA = \displaystyle \oint _{\Gamma } D_m\nabla F \cdot \mathbf{n} dS, \\ \quad \displaystyle \iint _{\chi } \nabla \cdot (D_m \nabla M) dA = \displaystyle \oint _{\Gamma } D_m\nabla M \cdot \mathbf{n} dS, \\ \quad \displaystyle \iint _{\chi } \nabla \cdot (D_g \nabla G) dA = \displaystyle \oint _{\Gamma } D_g \nabla G \cdot \mathbf{n} dS, \end{aligned}$$

where $\mathbf{n}$ is a normal vector pointing outwards the region $\chi$. For this particular estimate, let us consider that $\chi$ is isolated from the neighbor regions, i.e., that there are no mosquitoes entering or leaving $\chi$. Mathematically this is equivalent to $\nabla F \cdot \mathbf{n} = 0$, $\nabla M \cdot \mathbf{n} = 0$, and $\nabla G \cdot \mathbf{n} = 0$ on $\Gamma$.

Under these hypotheses, integrating System (1) in $\chi$, yields the following system of ODEs:

6 $$\begin{aligned} {\left\{ \begin{array}{ll} d_t {\mathbf{E}}&{}= \alpha \beta {\mathbf{F}}{\mathbf{M}}- e {\mathbf{E}}, \\ d_t {\mathbf{A}}&{}= e {\mathbf{E}}\left( 1 - \frac{{\mathbf{A}}}{K}\right) - (\mu _a + \eta _a){\mathbf{A}},\\ d_t {\mathbf{F}}&{}= r \eta _a {\mathbf{A}}- \mu _f {\mathbf{F}},\\ d_t {\mathbf{M}}&{}= (1-r) \eta _a {\mathbf{A}}- \mu _m {\mathbf{M}},\\ d_t {\mathbf{G}}&{}= L - \mu _{g} {\mathbf{G}}, \end{array}\right. } \end{aligned}$$

where all the constants are the same as in (1), except *L* which is the function representing the number of genetically modified mosquitoes released inside $\chi$ in a unit of time, and *K* is the carrying capacity of $\chi$ addressed in the next section. In System (6) we use bold variable names ${\mathbf{E}}$, ${\mathbf{A}}$, ${\mathbf{F}}$, ${\mathbf{M}}$, and ${\mathbf{G}}$ meaning a total population of the corresponding phase in $\chi$.

Next, we follow methodology from the literature^24,40^ and estimate the carrying capacity from the equilibrium conditions for different phases.

### Equilibrium point in the base case without genetically modified mosquitoes

In ODEs theory, an equilibrium point is a stationary solution (the one obtained equating the right side of equations (6) to zero)^41^. First, we find the non-trivial equilibria of System (6) for the particular case without genetically modified mosquitoes. Setting $L=0$, let us find $({\mathbf{E}}^*, {\mathbf{A}}^*, {\mathbf{F}}^*, {\mathbf{M}}^*, {\mathbf{G}}^*)$, such that $({\mathbf{E}}', {\mathbf{A}}', {\mathbf{F}}', {\mathbf{M}}', {\mathbf{G}}') = (0, 0, 0, 0, 0)$. After some calculations we arrive at ${\mathbf{G}}^* = 0$ and $\beta r(1-r) \eta _a^2 ({\mathbf{A}}^*)^2 - \beta r (1-r) \eta _a^2 K{\mathbf{A}}^* + (\eta _a + \mu _a) \mu _f \mu _m K = 0$. Isolating *K* we obtain the estimate for the total carrying capacity in a single grid block $\chi$:

7 $$\begin{aligned} K= \dfrac{\beta r(r-1) \eta _a^2 ({\mathbf{A}}^*)^2}{ \beta r (r-1) \eta _a^2 {\mathbf{A}}^* + (\eta _a + \mu _a) \mu _f \mu _m }. \end{aligned}$$

Solutions of the quadratic equation (7) can be found depending of the basic offspring number^42^

8 $$\begin{aligned} Q_0 = \dfrac{\beta r(1-r) \eta _a^2 K}{4(\eta _a + \mu _a) \mu _f \mu _m}. \end{aligned}$$

This number determines whether the analyzed population is growing, decreasing, or remains constant. Simple analysis leads us to the following result.

Proposition

- When $Q_0 < 1$, inside the variables’ definition domain (3), System (6) possesses only a trivial equilibrium $({\mathbf{E}},{\mathbf{A}},{\mathbf{F}},{\mathbf{M}},{\mathbf{G}}) = (0,0,0,0,0,0)$.
- If $Q_0 \ge 1$, inside the variables’ definition domain (3), System (6) possesses at least one admissible non-trivial equilibrium (${\mathbf{F}}^*>0$, ${\mathbf{M}}^{*}>0$, ${\mathbf{E}}^*>0$, and $0<{\mathbf{A}}^*<K$) given by
  9 $${\mathbf{A}}^{*} = K (1 + \sqrt{1 - 1/Q_0})/2,$$
  10 $${\mathbf{E}}^{*} = (\mu_a + \eta_a) K Q_0 (1 + \sqrt{1 - 1/Q_0})^{2}/e, $$
  11 $${\mathbf{F}}^{*} = K \eta_a(1 + \sqrt{1 - 1/Q_0})/(4\mu_f), $$
  12 $${\mathbf{M}}^{*} = K \eta_a(1 + \sqrt{1 - 1/Q_0})/(4\mu_f), $$
  13 $${\mathbf{G}}^{*} = 0. $$

In the current work, for simplicity, we consider that the equilibrium population of adult female mosquitoes in the region of interest is 10000, which corresponds to an average density $0.25\,\text {mosq.}/m^{2}$. As detailed in section Modeling, we consider that carrying capacities in the house blocks $K_h$, and in the streets, $K_s$ are related 1 : 5 ($K_h = 5 K_s$). Thus, we can estimate the adult female equilibrium population at each grid block using (11) and the portion of the domain corresponding to houses $\Omega _h$ and streets $\Omega _s$ (${\mathbf{F}}^* = \Omega _h {\mathbf{F}}^*_h + 0.2\, \Omega _s {\mathbf{F}}^*_s$). In the map we use in section Numerical Results, $\Omega _h = 26800\, \text {m}^2$ and $\Omega _s = 13200\, \text {m}^2$.

#### Remark 1

Notice that Eqs. (9)–(12) allow to use of data from different literature sources to estimate the coefficient *K*. For example, some authors^43,44^ collected the number of ovitraps in which the females laid eggs in a determined evaluated region. This data can be used to estimate ${\mathbf{E}}^*$. Another work^45^ shows the spatial distribution of *Ae. aegypti* and *Ae. albopictus* larval densities, which can be used to estimate ${\mathbf{A}}^*$. Other investigations^44^ presents the concentration of *Ae. aegypti* females, which can be used to estimate ${\mathbf{F}}^*$. The presented methodology allows comparing these experimental procedures.

#### Remark 2

In fact, System (6) possesses another root (different from (9)–(12)) with positive real part. However, as we will see in the next section, this root does not correspond to an attractor and does not influence the simulations.

#### Remark 3

Notice that System (6) with $L=0$ always possess an equilibrium in $(E,A,F,M,G) = (0,0,0,0,0,0)$. Although this equilibrium is also an attractor for System (6), its attraction basin is small. In other words, if the initial quantity of mosquitoes in all phases is sufficiently low, they tend to zero with time. As in this paper we are interested in the case when vector control is necessary, we focus our analysis on the cases with non-trivial equilibria.

### Equilibrium points considering the application of genetically modified male mosquitoes

As will be shown in the next sections, the frequency of release of genetically modified male mosquitoes is an essential factor in designing strategies to combat *Ae. aegypti*. To estimate the equilibrium accounting for this factor, let us follow a common mathematical approach^46^ and assume that the same number of genetically modified mosquitoes is released every day, i.e., *L* is a positive constant. Following the same procedure as above, we find solutions $({\mathbf{E}}^\#, {\mathbf{A}}^\#, {\mathbf{F}}^\#, {\mathbf{M}}^\#, {\mathbf{G}}^\#)$, such that $({\mathbf{E}}', {\mathbf{A}}', {\mathbf{F}}', {\mathbf{M}}', {\mathbf{G}}') = (0, 0, 0, 0, 0, 0)$:

14 $$\begin{aligned} ({\mathbf{A}}^\#)^3 - K ({\mathbf{A}}^\#)^2 + b K^2 ({\mathbf{A}}^\#) + bcK = 0, \qquad \text {where} \qquad b = (\mu _a + \eta _a)\dfrac{\mu _f \mu _m }{(1-r)r\beta \eta _a^2}, \qquad c = \dfrac{\mu _m L}{(1-r)\eta _a \mu _g}. \end{aligned}$$

To solve the cubic equation (14) we apply a particular form^47^ of Cardano’s equation in terms of the discriminant

15 $$\begin{aligned} \phi = \dfrac{1}{27} \left( b K-\dfrac{K^2}{3} \right) ^3 + \dfrac{1}{4} \left( -\dfrac{2K^3}{27} + \dfrac{K^2 b}{3} + bcK \right) ^2. \end{aligned}$$

The first root of (14) is:

16 $$\begin{aligned} {\mathbf{A}}^\#_1 = \root 3 \of {\dfrac{2K^3}{54} - \dfrac{K^2b}{6} - \dfrac{c}{2} + \sqrt{\phi }} + \root 3 \of {\dfrac{2K^3}{54} - \dfrac{K^2b}{6} - \dfrac{c}{2} - \sqrt{\phi }} + \dfrac{K}{3}. \end{aligned}$$

To obtain the other roots (${\mathbf{A}}^\#_2$ and ${\mathbf{A}}^\#_3$) we divide the cubic (14) by ${\mathbf{A}}^\#_1$ and solve the resulting quadratic equations. As the expressions for ${\mathbf{A}}^\#_2$ and ${\mathbf{A}}^\#_3$ are extensive and do not have direct applications, we do not present them here.

- For $\phi > 0$ all three roots are not admissible as neither of them satisfy (3): ${\mathbf{A}}^\#_3$ is negative; ${\mathbf{A}}^\#_1$ and ${\mathbf{A}}^\#_2$ are complex numbers.
- If $\phi < 0$, (14) possesses three real roots: ${\mathbf{A}}^\#_3$ is negative; ${\mathbf{A}}^\#_1$ and ${\mathbf{A}}^\#_2$ are positive satisfying (3).

**Figure 1:**
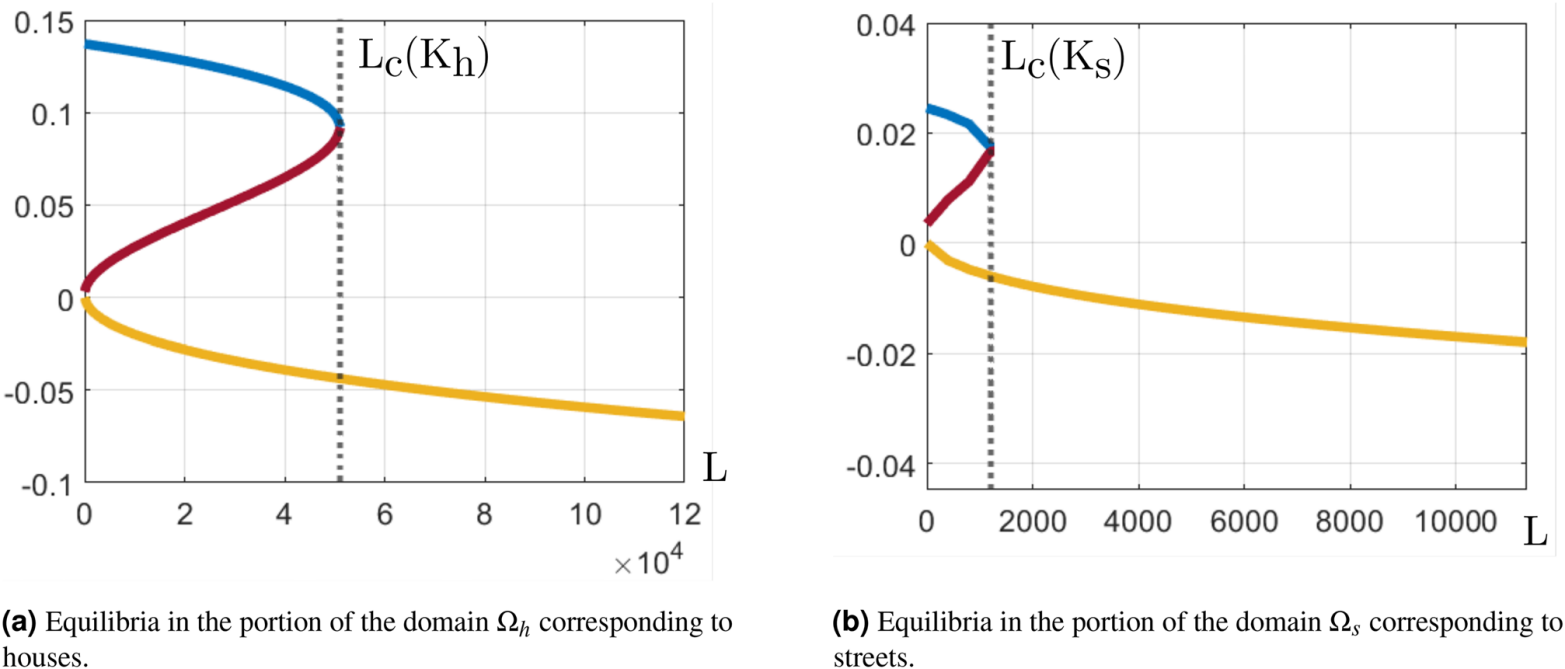
Three equilibria ${\mathbf{A}}^\#_1$ (blue line), ${\mathbf{A}}^\#_2$ (red line), and ${\mathbf{A}}^\#_3$ (yellow line) of the System (6) as function of *L*. The critical values $L_c(K)$ are calculated using Eq. (17) at $K_h$, and $K_s$ - carrying capacities of the house blocks and streets.

Figure 1 shows all three roots ${\mathbf{A}}^\#_1$, ${\mathbf{A}}^\#_2$, and ${\mathbf{A}}^\#_3$ for different values of *L* calculated in house grid blocks (using $K_h$) and street grid blocks (using $K_s$) using parameter values from Table 1. Notice that in both panels there is a critical value $L_c$, when $\phi =0$. It can be obtained analytically:

17 $$\begin{aligned} L_c(K) = \dfrac{(1-r) \eta _a \mu _g}{bK \mu _m} \left( \sqrt{-\dfrac{4}{27}\left( bK - \dfrac{K^2}{3} \right) ^3} + \dfrac{2K^3}{27} - \dfrac{K^2 b}{3}\right) . \end{aligned}$$

Beyond different types of equilibria, the one that interests us here is an attractor, which has the following property. All solutions tend to this point as time tends to infinity for any initial data in the attractor’s neighborhood. In particular, this type of equilibria happens when all the eigenvalues of the Jacobian matrix of the functions in the right side of Eq. (6) possess negative real parts, details can be found here^41^. For the equilibria plotted in Fig. 1, ${\mathbf{A}}^\#_1$ is an attractor, ${\mathbf{A}}^\#_2$ is a saddle, ${\mathbf{A}}^\#_3$ is an attractor when $L=0$ and a saddle when $L>0$. Although we did not prove this property mathematically here, it was studied for similar models in the literature^20,42,48^ and we also verified it numerically in all our simulations. Comparing ${\mathbf{A}}^\#_1$ to ${\mathbf{A}}^\#_2$, as the former is an attractor and the latter is a saddle, it is natural to expect that the corresponding simulation of the original System (1) tends to ${\mathbf{A}}^\#_1$. On the other hand, equilibrium ${\mathbf{A}}^\#_3$ stays outside the definition domain given in Eq. (3), which we proved to be invariant in section Modeling. Thus, it is natural to expect that the corresponding solution of System (1) tends to zero as it can not cross the invariant domain’s boundary.

**Figure 2:**
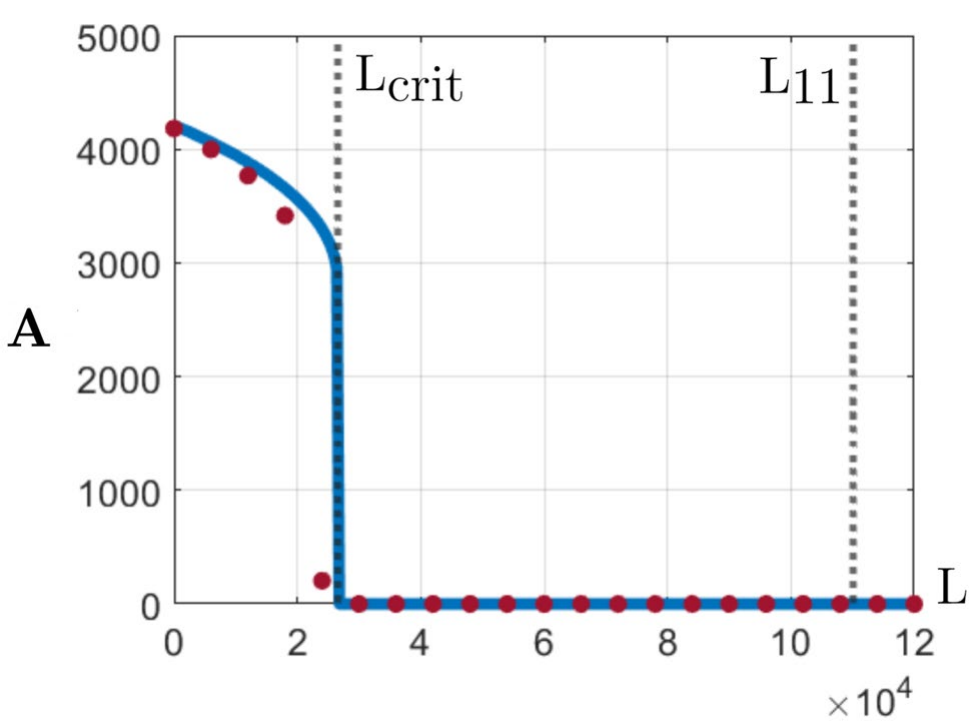
The expected total aquatic phase population equilibrium (the sum of all $\mathbf {A}^\#_1$ at each grid block) as a function of *L* (daily release of genetically modified mosquitoes) is indicated by the solid blue curve. Each red dot (“”) corresponds to numerical simulation result for the system (1). The value $L_{crit}$ is calculated using Eq. (18) and $L_{11}$ is 11 times the equilibrium of the adult female population.

Summarizing the analysis above, we can construct the expected total mosquitoes population as a function of the daily release of GM mosquitoes by adding the ${\mathbf{A}}^\#_1$ functions (where both exist) and setting the expectation as zero, when there is only one (${\mathbf{A}}^\#_3<0$) equilibrium. The result is plotted in Fig. 2. Notice that there is a critical value for the daily release of GM mosquitoes, beyond which the equilibrium population is zero. This critical value can be estimated by applying (17) to the weighted average of both carrying capacities:

18 $$\begin{aligned} L_{crit} = L_c\left( \dfrac{n_h K_h +n_s K_s}{n_h + n_s} \right) , \end{aligned}$$

where $n_h$ and $n_s$ represent the quantities of house and street grid blocks, respectively. Following the literature^30^, in the next section, we consider the released GM mosquito population eleven times the size of the female phase equilibrium. This amount corresponds to 109983 GM mosquitoes per day or 3299500 GM mosquitoes per 30-day period indicated by $L_{11}$ in Fig. 2.

## Numerical results and discussion

In the previous section, we investigated model’s asymptotic solutions using analytical tools. While this approach allows us to obtain qualitative data on the population dynamics, the quantitative results need the solution (each time and space point) of the non-linear system of partial differential equations (1), which is only possible to achieve through direct numerical simulations. The employed numerical method is described in section Methods; below, we discuss numerical results.

**Figure 3:**
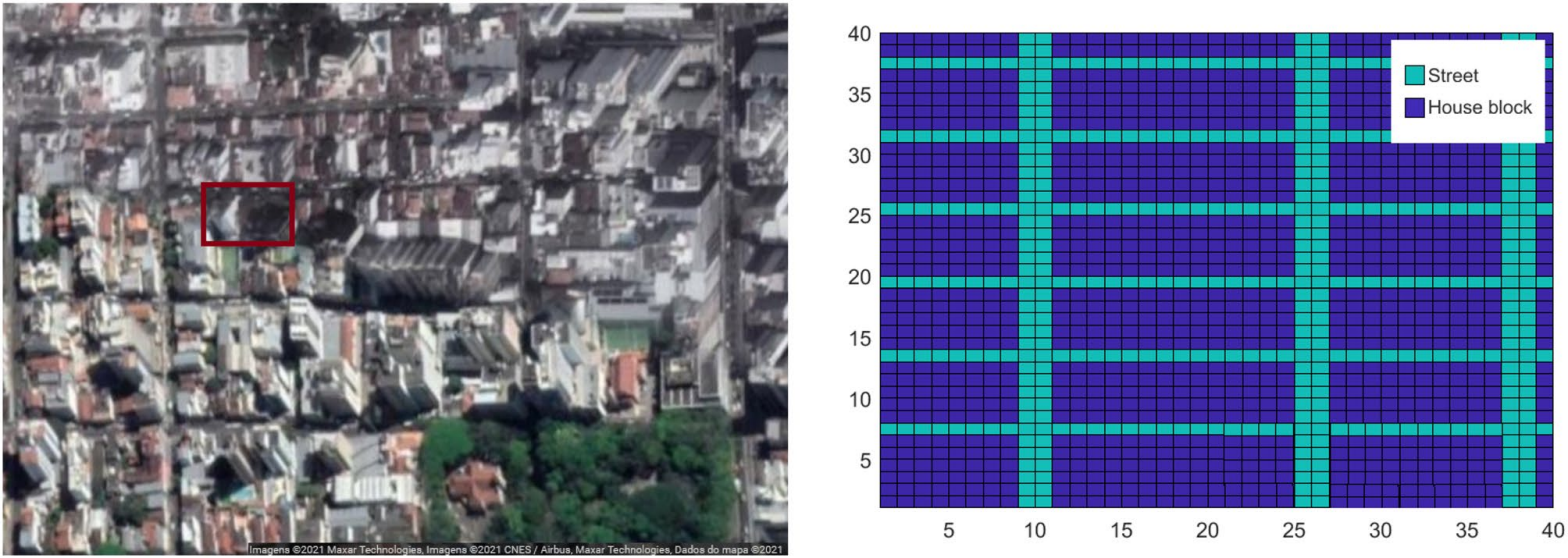
On the left, satellite view of a neighborhood of Juiz de Fora - Brazil obtained from Google Maps. On the right, numerical representation of the map as a $40\times 40$ matrix. On the left panel, we highlighted a small area where the localized liberation of genetically modified mosquitoes happens as described on the Numerical results section.

All the experiments are run on the same heterogeneous map^21^ representing a central neighborhood ($200\,\text {m} \times 200\,\text {m}$) located in Juiz de Fora, Brazil, as depicted in the left panel in Figure 3. This domain is discretized in $40\times 40$ grid blocks, as depicted in the right panel in Figure 3 separating street environment from house blocks. Parameter values used in the simulations are summarized in Table 1. Using (7) we estimate the carrying capacity in each house grid block as $K_h= 3.6876$ and in each street grid block as $K_s= 0.7375$, which correspond to $k_h= 0.1402$ and $k_s=0.0280$ #mosquitoes/m$^2$.

Section Analytical Results states that we need an equilibrium population of one of the phases to estimate all the other phases and the carrying capacity using equations (9)–(13). Estimating mosquito density is challenging^49^. In the literature^50–53^, one can find estimates that the equilibrium pupae population is proportional to population density in the area, varying from 0.12 to 0.99 pupae/person. Using population density in Juiz de Fora city central area and equations (9)–(13), one can estimate the female equilibrium population. This paper assumes the initial mosquito density to be 0.25 mosq./m$^2$ (inside the above interval), equivalent to 10.000 mosquitoes in our simulation domain of 40.000 m$^2$.

### The impact of heterogeneity in the spatial population dynamics

**Figure 4:**
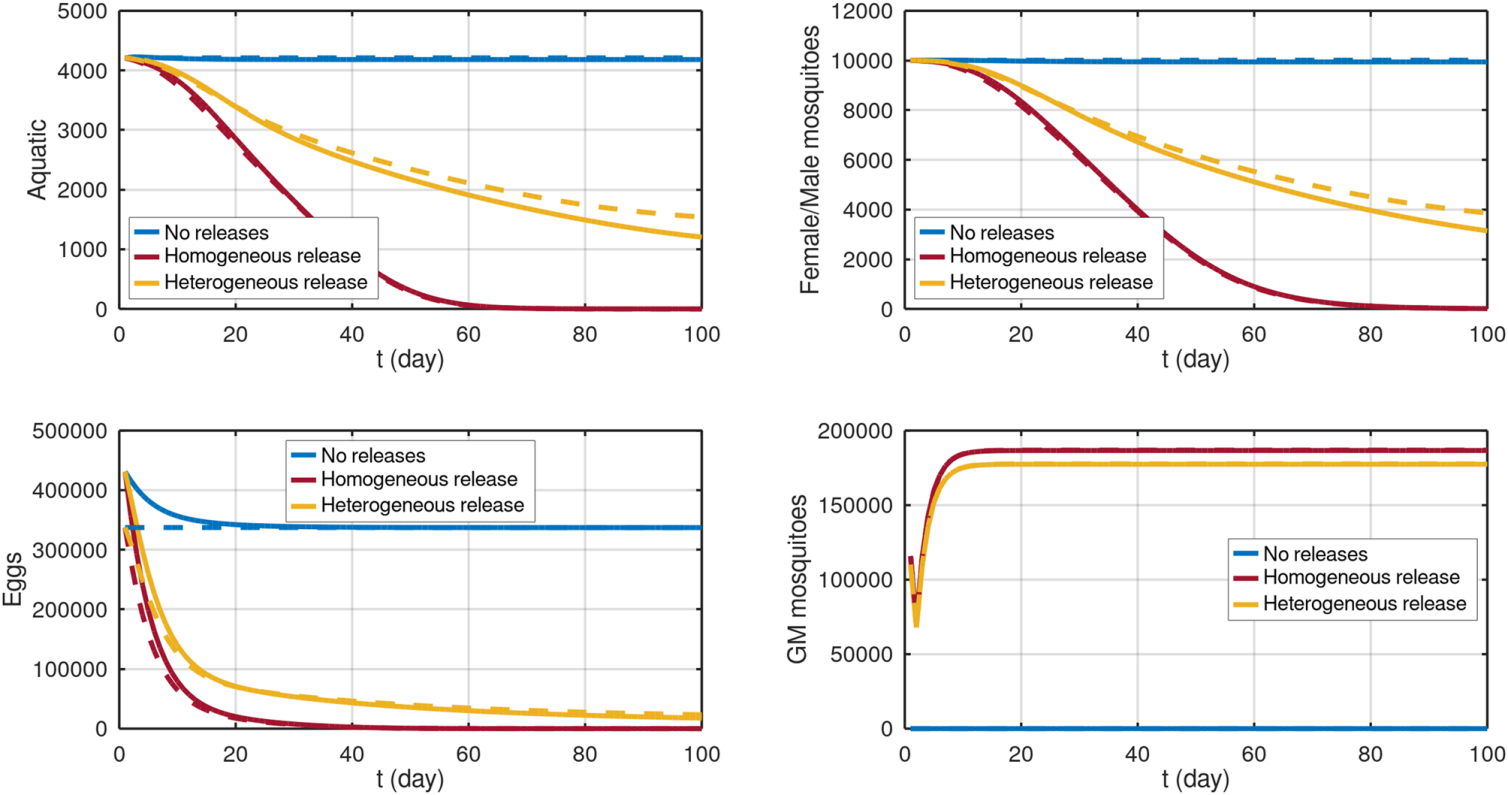
Populations density in different release strategies. The continuous lines represent the dynamics on a heterogeneous map (carrying capacity depend on location), and the dotted lines refer to the homogeneous map (same carrying capacity on all map). The homogeneous release considers that the GM mosquitoes are uniformly released on the map. The heterogeneous release means that this release happens inside the region highlighted in the left panel of Figure 3. Both strategies release the same amount of mosquitoes.

Genetically modified male mosquitoes are not liberated in the hole domain homogeneously but in some points of the city^30^. This feature can not be modeled based on an ordinary differential equation dealing with the total population. To exemplify this issue, let us assume that genetically modified mosquitoes are liberated in a small central area indicated by the red rectangle in the left panel of Fig. 3. We recall that the adult female mosquitoes’ equilibrium population in this region is considered to be 10.000. As detailed in section Modeling, the number of GM mosquitoes liberated in a day is 11 times the female population, yielding 110.000 GM mosquitoes liberated daily in the small area of $943.71\,\text {m}^2$ (indicated by the red rectangle in the left panel of Fig. 3) equivalent to a density of 116 mosq./m$^2$ inside this area. The 100-day simulation results are indicated by the solid orange line in Fig. 4. The starting point to all simulations in Fig. 4 was chosen as the equilibrium conditions given by equations (9)–(12).

In order to see the impact of the localized GM mosquitoes release (heterogeneous release), we run our model considering the same total amount of GM mosquitoes is liberated in an entire domain (homogeneous release), i.e., daily release of 0.0029 1/m$^2$ mosquitoes. The 100-day simulation results are indicated by the solid red line in Fig. 4.

Finally, we ran a control simulation without GM mosquitoes. The 100-day simulation results are indicated by the solid blue line in Fig. 4.

All simulations above were performed on the heterogeneous map divided into house and street grid blocks, as depicted in the right panel in Fig. 3, using parameter values from Table 1. To understand the impact of this assumption, we repeat the three simulations above considering homogeneous map, i.e., considering that the street and house grid blocks have the same carrying capacity (weighted average carrying capacity of both). The 100-day simulation results are indicated by the dashed lines in Fig. 4.

Let us discuss the simulation results presented in Fig. 4. First of all, the simulation results for a heterogeneous map without GM mosquitoes (solid blue lines) remain close to the equilibrium calculated using equations (9)–(12), validating the analytical estimates presented in the previous section.

Secondly, the impact of considering the heterogeneity in carrying capacity coefficient (solid and dashed yellow curves) corresponds to a difference of up to $8\%$ of the initial population after 100 days for aquatic and mobile phases. These results, at least for parameter values considered in this work, give an idea of how far a traditional total population approach using ODEs stays from the heterogeneous modeling.

Finally, the situation is different when comparing realistic localized GM mosquitoes release with a homogeneous one (see red and yellow curves). After 100 day simulation, the difference between total populations is approximately $29\%$ of the initial population in aquatic and mobile phases, and the maximum difference of $44\%$ happens around 60 days. At least for parameter values considered in this work, we conclude that the traditional total population approach using ODEs would not represent this phenomenon accurately.

### Validation of the analytical results

The most important result of this paper is the formula (17) estimating the critical amount of GM released mosquitoes. In order to validate it, we run a series of simulations of the system (1) on a heterogeneous map (separating houses and streets), varying the total amount of GM mosquitoes homogeneously released daily (*L*). The total aquatic population at the end of 300-day simulations are indicated by the red dots on Fig. 2, and represents the equilibrium that each simulation achieved. While this case corresponds to daily GM mosquitoes’ release, as will be shown in the next section, these results apply to other low release frequencies.

As we have shown, there exists a critical $L_{crit}$ value for GM released mosquitoes that Equation (18) can predict before solving the model. The total population of mosquitoes tends to zero or is not qualitatively affected depending on if the amount of released mosquitoes is above or below the critical value. Although the value of $L_{crit}$ can change considering different parameter values, our findings point out that this parameter needs to be taken into account when planning GM mosquitoes release aiming vector control. In particular, this technique can be affected by GM mosquitoes’ early death, yielding to unsuccessful application.

### The frequency of application of genetically modified mosquitoes

**Figure 5:**
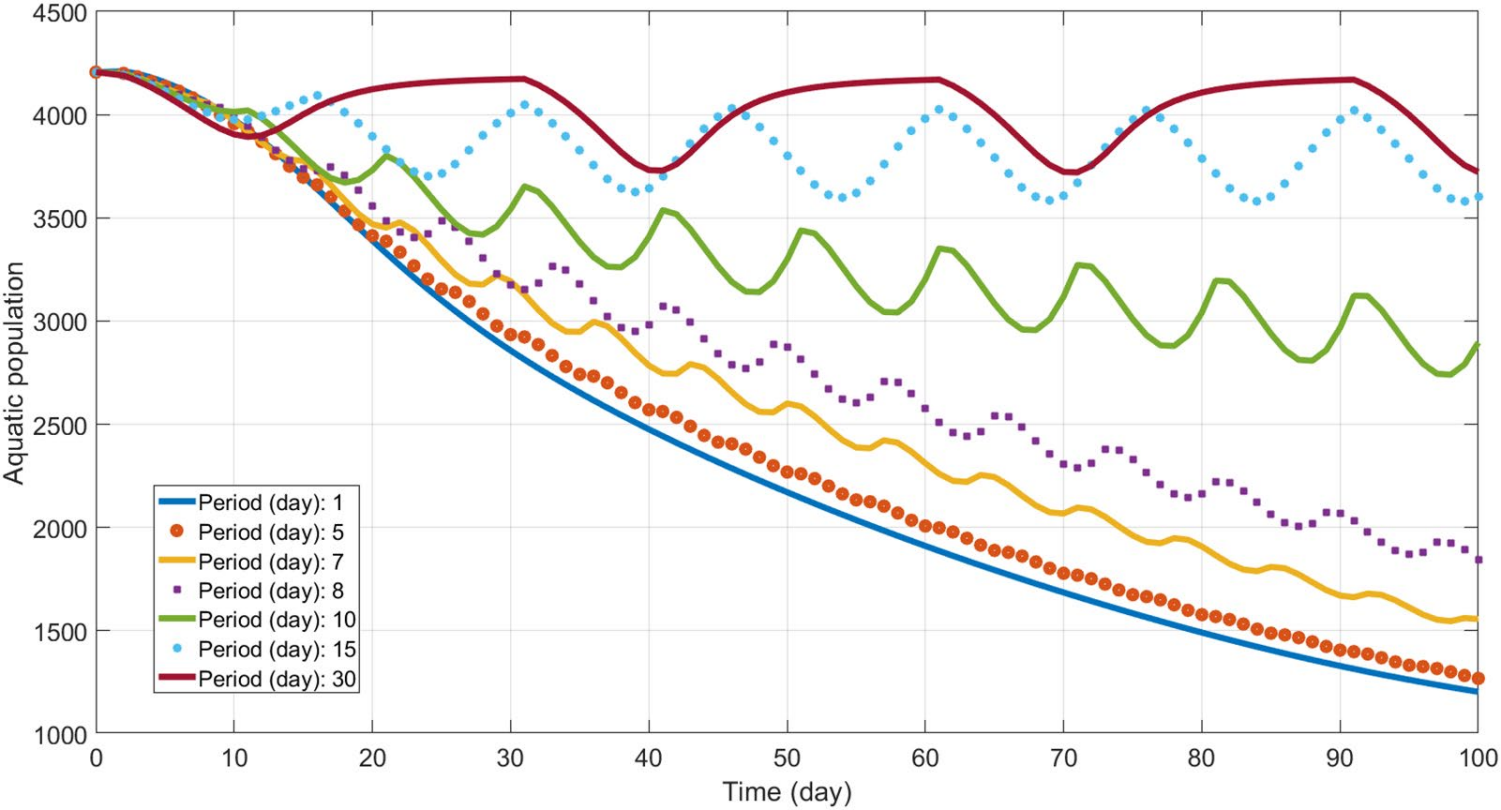
Temporal evolution of the aquatic phase population during 100 days with different release periods. The mosquitoes were liberated in a smaller central area highlighted on the left panel of Fig. 3.

The next question we address in this work is how the periodicity of the GM mosquitoes release can impact the temporal evolution of the population dynamics in the heterogeneous region. Maintaining the total amount of released GM mosquitoes fixed (following the literature^30^, we consider the released GM mosquito population eleven times the size of the female phase equilibrium, which corresponds to 109983 GM mosquitoes per day or 3299500 GM mosquitoes per 30 day period) we vary the release frequency from once a day to once in 30 days. Simulation results for the aquatic phase are shown in Fig. 5. All simulations start as $A=4206$ corresponding to equilibrium population ${\mathbf{A}}^*$ from Eq. (9).

**Figure 6:**
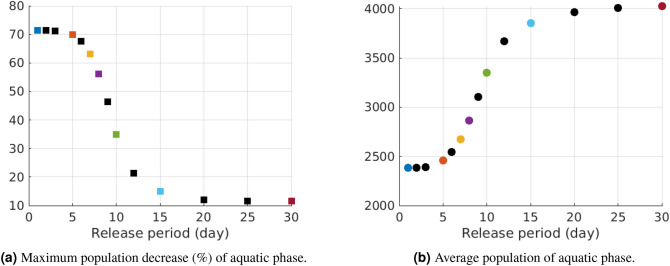
The impact of the frequency of GM mosquitoes release in 100 days simulations. The results plotted with the colored points correspond to the simulations presented in Fig. 5. The total amount of GM mosquitoes in 30 day period is fixed in all simulations.

In order to better understand the results shown in Fig. 5 we compare the maximum aquatic population decrease in the first 100 days of simulations for different release periods (see Fig. 6a) and an average aquatic population in the first 100 days of simulation (see Fig. 6b). Although in Figs. 5 and 6 we plot the results for the aquatic population, other phases present similar behaviour.

- From the simulation results plotted in Figs. 5 and 6 it is clear that there is an abrupt change in behavior around 7–8 day frequency, which is close to the release frequency found in the literature^12^. Below this value, the mosquitoes population tends to zero, while above it tends to zero unacceptably slow, or there is no qualitative decrease. Notice that this value coincides a development cycle of mosquitoes from an egg to the adult emergence under favorable conditions^7^.
- The results exhibited in Figs. 5 and 6 validate analytical estimated and numerical simulation presented in the previous section for daily GM mosquitoes release as for small frequencies, the qualitative trends are the same.
- Simulation results plotted in Figs. 5 and 6 point that even a sufficient total amount of GM released mosquitoes can lead to unfavorable results if applied in time intervals larger than 7 days.

## Conclusion

- We present a model that describes the spatial dynamics of the *Ae. aegypti* mosquito population in the presence of the GM males. The model is composed of partial differential equations and is based on the assumption of the dispersal capacity of male and female mosquitoes. The model presents some mathematical improvements compared to the literature as it is based on smooth functions allowing deeper mathematical analysis. We also showed that the model’s variable domain is invariant for time evolution, guaranteeing that the model will not result in meaningless solution profiles. Moreover, this model relies on a few parameters which, as we show in this work, can be obtained or estimated from the literature. We stress that this model is limited to the ideal conditions as it does not consider the parameter dependence on temperature, food availability, and humidity.
- We presented a detailed framework for making a parallel between spatial population dynamics (modeled through PDEs) and total population dynamics (modeled by using ODEs), preserving the main modeling properties. This technique allows investigating of the proposed model mathematically and finding stationary (equilibrium) solutions.
- The analysis presented in this work allowed us to show that there exists a critical number of GM released mosquitoes above which the population of adult mosquitoes tends to zero, see Fig. 2. If the total amount of released GM mosquitoes is below this critical number, the total population does not decrease significantly. On the other hand, when releasing more GM males, the total population of mosquitoes in all phases tends to zero.
  We presented an analytical formula (17)–(18) for this critical value and validated these findings through numerical simulations for the two-dimensional model in a heterogeneous map. This formula indicates that the total amount of released GM males is a critical parameter to be taken into account when planning or controlling the application of this vector control technique.
  Although we estimated parameter values from the literature, limiting quantitative conclusions, the quantity of GM males reported^30^ in applications (11 times the population) is adequate for *Ae. aegypti* control, see Fig. 2.
- The impact of heterogeneity in carrying capacity for the analyzed parameter values is not pronounced (up to 8% of the initial population after 100 days). On the other hand, the effect of localized GM mosquitoes release is significant (up to 44% of the initial population after 60 days). These results point to the limitations in total population modeling and the importance of spatial modeling.
- The investigation of different release frequencies for the analyzed parameter values evidences a maximum GM mosquitoes release time interval. Releasing larger quantities of mosquitoes in larger intervals does not affect the wild mosquitoes population significantly. The critical time interval for our simulations is approximately 7 days, which is close to the release periodicity found in the literature^12^.
- As a final conclusion, we state that the correct and efficient application of GM mosquitoes should consider the critical amount of these mosquitoes and the maximum allowed period between releases.

## Methods

### Finite volume

The equations describing the population dynamics of *Ae. aegypti* have been discretized using an implicit FVM^54^ detailed next. The domain is given by $\Omega = [0, L] \times [0, L]$. In order to rewrite the System (1) in the weak form, we integrate it in a control volume $\omega _{ij} \subset \Omega$:

19 $$\begin{aligned} \displaystyle \iint _{\omega _{ij}}\frac{\partial E}{\partial t} dx dy= & {} \displaystyle \iint _{\omega _{ij}}(\alpha (M,MG) \beta F M -e E) dx dy. \end{aligned}$$

20 $$\begin{aligned} \displaystyle \iint _{\omega _{ij}}\frac{\partial A}{\partial t} dx dy= & {} \displaystyle \iint _{\omega _{ij}}\left[ e \left( 1 - \dfrac{A}{k} \right) E -(\eta _a+{\mu _a})A\right] dx dy, \end{aligned}$$

21 $$\begin{aligned} \displaystyle \iint _{\omega _{ij}} \frac{\partial F}{\partial t} dx dy= & {} \displaystyle \iint _{\omega _{ij}} \nabla \cdot (D_f \nabla F) dx dy + \displaystyle \iint _{\omega _{ij}}(r \eta _a A - \mu _f F) dx dy, \end{aligned}$$

22 $$\begin{aligned} \displaystyle \iint _{\omega _{ij}} \frac{\partial M}{\partial t} dx dy= & {} \displaystyle \iint _{\omega _{ij}} \nabla \cdot (D_m \nabla M) dx dy + \displaystyle \iint _{\omega _{ij}}((1-r) \eta _a A - \mu _m M) dx dy, \end{aligned}$$

23 $$\begin{aligned} \displaystyle \iint _{\omega _{ij}} \frac{\partial G}{\partial t} dx dy= & {} \displaystyle \iint _{\omega _{ij}} \nabla \cdot (D_m \nabla G) dx dy + \displaystyle \iint _{\omega _{ij}}( - \mu _{g} G + l) dx dy, \end{aligned}$$

Considering $\omega _{ij}$ as a cell centered in $(x_i , y_j )$, we solve each integral separately. For the left side of the System (19)–(23), taking one of the functions arbitrarily as *U*, it follows:

24 $$\begin{aligned} \displaystyle \int _{y_{j-1/2}}^{y_{j + 1/2}} \displaystyle \int _{x_{i-1/2}}^{x_{i + 1/2}} \dfrac{\partial U(x,y,t)}{\partial t} dx dy \approx \Delta x \Delta y \dfrac{U_{i,j}^{n+1} - U_{i,j}^{n}}{\Delta t}, \end{aligned}$$

where $$U(x_i,y_j,t_n) = U_{i,j}^n$$.

For the second term in (21)–(23) (diffusion term), first consider the derivative only in the X direction:

25 $$\begin{aligned}&\displaystyle \int _{y_{j-1/2}}^{y_{j + 1/2}} \displaystyle \int _{x_{i-1/2}}^{x_{i + 1/2}} \dfrac{\partial }{\partial x} \left( D_{f} \dfrac{\partial F}{\partial x} \right) dx dy \approx \Delta y\left[ D_{f}^{i+1/2,j} \left( \dfrac{F^n_{i+1,j} - F^n_{i,j} }{\Delta x} \right) - D_{f}^{i-1/2,j} \left( \dfrac{ F^n_{i,j} - F^n_{i-1,j} }{\Delta x} \right) \right] , \end{aligned}$$

where $$D_{f}^{i\pm 1/2,j} = (D_f(x_i,y_j) + D_f(x_i \pm 1,y_j)) / 2$$.

Using a similar calculation for the Y direction and adding both equations for X and Y directions, we obtain the second term in (21) (diffusion term) and the process can be repeated for equations (22) and (23). The integral of each source term is approximated as follows:

26 $$\begin{aligned}&\displaystyle \int \displaystyle \int _{\omega _{ij}}(\alpha \beta F M - e E) dx dy \approx (\alpha ^n_{ij} \beta F^n_{ij} F^n_{ij} - e E^n_{ij}) \Delta x \Delta y, \end{aligned}$$

27 $$\begin{aligned}&\displaystyle \int \displaystyle \int _{\omega _{ij}} \left[ e \left( 1 - \dfrac{A}{k}\right) E - (\mu _a + \eta _a)A \right] dx dy \approx \left[ e \left( 1 - \dfrac{A^n_{ij}}{k} \right) E^n_{ij} - (\mu _a + \eta _a)A^n_{ij} \right] \Delta x \Delta y, \end{aligned}$$

28 $$\begin{aligned}&\displaystyle \int \displaystyle \int _{\omega _{ij}}(r \eta _a A - \mu _f F) dx dy \approx (r \eta _a A^n_{ij} - \mu _f F^n_{ij}) \Delta x \Delta y, \end{aligned}$$

29 $$\begin{aligned}&\displaystyle \int \displaystyle \int _{\omega _{ij}}((1-r) \eta _a A - \mu _m M) dx dy \approx ((1-r) \eta _a A^n_{ij} - \mu _m M^n_{ij}) \Delta x \Delta y, \end{aligned}$$

30 $$\begin{aligned}&\displaystyle \int \displaystyle \int _{\omega _{ij}}(- \mu _{g} G + l) dx dy \approx (- \mu _{g} G^n_{ij} + l) \Delta x \Delta y. \end{aligned}$$

With the presented approximations, we use a Crank–Nicolson scheme already detailed in previous work on a smaller model^24^. The simulation consists in solving a nonlinear system for $E^{n+1}$, $A^{n+1}$, $F^{n+1}$, $M^{n+1}$ and $G^{n+1}$ at each time step to calculate the population distribution of each phase. We use a time step equal to 1 entire day. Stability and convergence details of this type of method have been extensively studied in the literature^54–56^.
